# Prevalence of Cardiovascular Events and Their Risk Factors in Patients With Chronic Obstructive Pulmonary Disease and Obstructive Sleep Apnea Overlap Syndrome

**DOI:** 10.3389/fcvm.2021.694806

**Published:** 2021-07-14

**Authors:** Manyun Tang, Yunxiang Long, Shihong Liu, Xin Yue, Tao Shi

**Affiliations:** ^1^Arrhythmia Unit, Department of Cardiovascular Medicine, The First Affiliated Hospital of Xi'an Jiaotong University, Xi'an, China; ^2^Department of Hepatobiliary Surgery, The First Affiliated Hospital of Xi'an Jiaotong University, Xi'an, China; ^3^East Unit, Department of Cardiovascular Medicine, The First Affiliated Hospital of Xi'an Jiaotong University, Xi'an, China; ^4^Department of Cardiology, Cedars-Sinai Medical Center, Smidt Heart Institute, Los Angeles, CA, United States; ^5^Department of Cardiovascular Surgery, The First Affiliated Hospital of Xi'an Jiaotong University, Xi'an, China

**Keywords:** chronic obstructive pulmonary disease, risk stratification, cardiac rhythm abnormalities, obstructive sleep apnea, overlap syndrome, cardiovascular events

## Abstract

**Rationale:** Chronic obstructive pulmonary disease (COPD) and obstructive sleep apnea (OSA) have been identified as independent risk factors for cardiovascular diseases. However, the impact of COPD and OSA overlap syndrome (OS) on cardiovascular outcomes remains to be elucidated.

**Objective:** To determine the prevalence of cardiovascular events and their risk factors in OS patients.

**Methods:** Seventy-four patients who had OS between January 2015 and July 2020 were retrospectively enrolled, and 222 COPD-only patients and 222 OSA-only patients were pair-matched for age and sex from the same period and served as the OS-free control group. The prevalence rates of coronary heart disease (CHD), arrhythmia, heart failure, and pulmonary arterial hypertension (PAH) were compared among the three groups, and multivariable logistic regression models were used to screen the risk factors for specific cardiovascular events.

**Results:** OS patients had higher prevalence rates of heart failure (10.8 vs. 0.5 and 1.4%, respectively) and PAH (31.1 vs. 4.5 and 17.1%, respectively) than those with OSA alone or COPD alone (all *P* < 0.01). The CHD prevalence was also significantly higher in the OS group than in the COPD-alone group (25.7 vs. 11.7%, *P* < 0.01). There was no significant difference in the prevalence of arrhythmia among the three groups (20.3, 22.5, and 13.1%, respectively, *P* > 0.05). In OS patients, risk factors for CHD included hypertension, diabetes, body mass index, lactate dehydrogenase level, and tidal volume; risk factors for heart failure included diabetes, partial pressure of oxygen, partial pressure of carbon dioxide, maximum ventilatory volume, and neutrophilic granulocyte percentage; and risk factors for PAH included minimum nocturnal oxygen saturation, partial pressure of carbon dioxide, and brain natriuretic peptide and lactate dehydrogenase levels.

**Conclusions:** OS patients have a higher prevalence of cardiovascular events, which is associated with hypoxemia, hypercapnia, and impaired lung function in these patients.

## Introduction

Chronic obstructive pulmonary disease (COPD) and obstructive sleep apnea (OSA) are among the most prevalent chronic diseases and represent a major reason for health-care utilization, imposing a heavy health burden worldwide (1–3). The prevalence of COPD in American adults is estimated to be 13.9% (4, 5) compared with 13.7% in Chinese adults aged 40 years or older (6), and the incidence of OSA in adults is close to 9–26% (7). Existing epidemiological data suggest that overlap syndrome (OS), which refers to the coincidence of both COPD and OSA (8), is not a common disease in the general population (incidence ranges from 1 to 3.6%), but patients who already have COPD or OSA exhibit a significant increase in the incidence of overlap syndrome (2.9–65.9%) (9).

COPD is characterized by incomplete reversible airflow obstruction that increases the risk of cardiovascular disease through increased sympathetic nervous activity and persistent low-grade systemic inflammation (10, 11). Obstruction of the upper airway during sleep causes OSA, which leads to periodic apnea and hypopnea, resulting in nocturnal hypoxemia. Patients with OSA usually complain of severe snoring, problems with being awakened by wheezing or suffocation, and daytime sleepiness (12, 13). OSA has also been recognized as a risk factor for cardiovascular diseases. Possible pathophysiological mechanisms include transient and repeated oxygen desaturation, which could induce oxidative stress, systemic inflammation, endothelial dysfunction, and autonomic dysfunction (14).

Under the synergistic effect of COPD and OSA, patients with OS have lower nocturnal oxygen saturation, more profound oxidative stress, more serious systemic inflammation, more severe vascular endothelial dysfunction, and accelerated atherosclerosis (15–17), all of which are believed to be strong risk factors for cardiovascular disease. It is well known that COPD and OSA are independent risk factors for cardiovascular events. However, real-world evidence concerning the prevalence and related risk factors for cardiovascular diseases in patients with COPD and OSA overlap syndrome has rarely been reported. Therefore, this study aimed to evaluate the prevalence of specific cardiovascular events and their risk factors in patients with COPD and OSA overlap syndrome to help improve the management of these patients.

## Methods

### Study Design

This is a cross-sectional study, and anonymized clinical data were collected from the Biobank of the First Affiliated Hospital of Xi'an Jiaotong University from January 2015 to July 2020. The Ethics Committee of the First Affiliated Hospital of Xi'an Jiaotong University approved this study (no. XJTU1AF2020LSK-187), and informed consent was obtained. All methods were carried out in accordance with the relevant guidelines and regulations according to the principles expressed in the Declaration of Helsinki.

### Subjects

**Figure 1:**
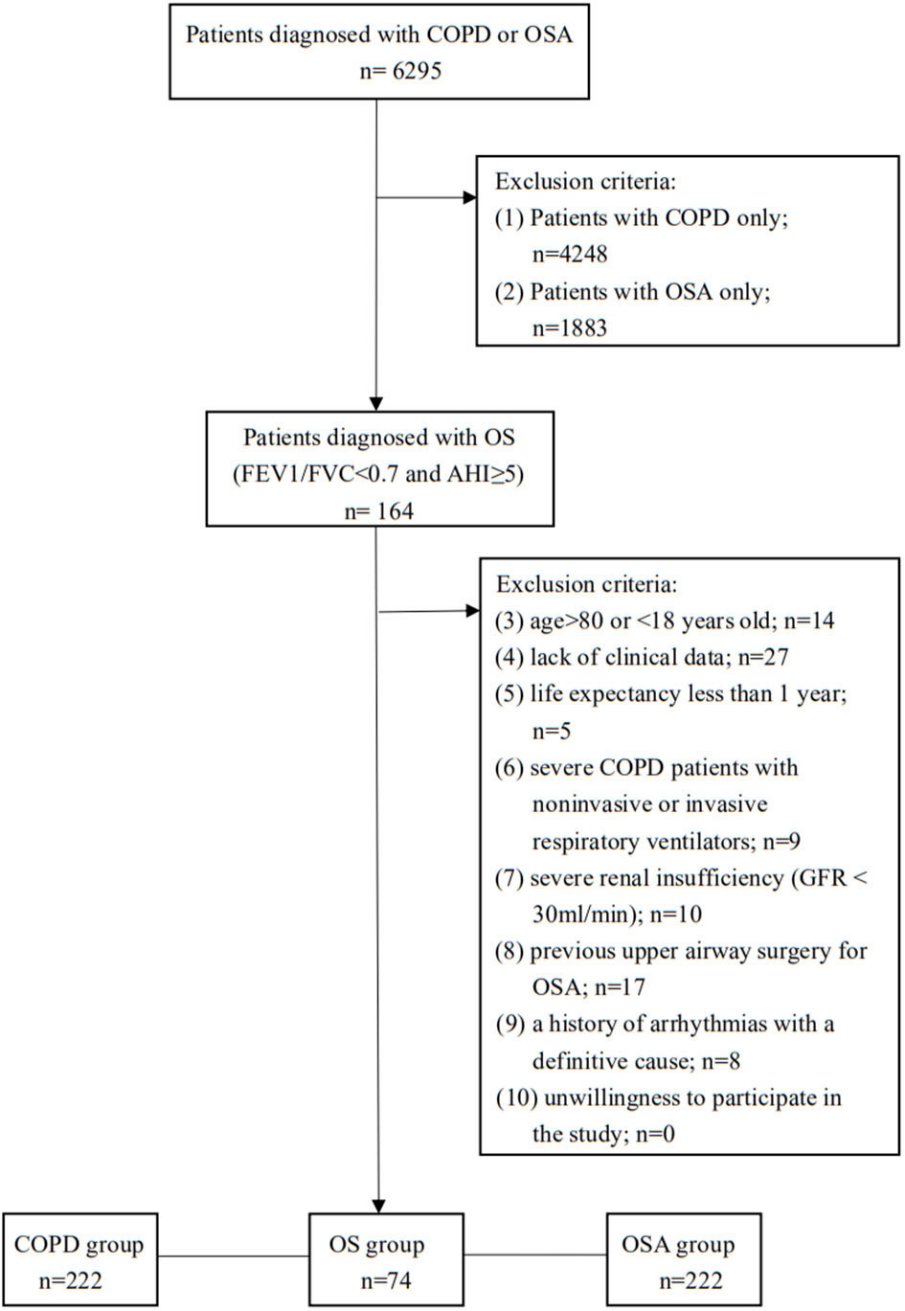
Study protocol. OS, overlap syndrome; OSA, obstructive sleep apnea; COPD, chronic obstructive pulmonary disease; AHI, apnea-hypopnea index; FEV1/FVC, forced expiratory volume in 1 s/forced vital capacity.

The medical records of all patients who were diagnosed with COPD or OSA were retrospectively analyzed. The diagnoses were based on clinical expert consensus documents. COPD should have been considered in any patient with dyspnea, chronic cough or sputum production, a history of exposure to risk factors for the disease, and a post-bronchodilator forced expiratory volume in 1 s (FEV1)/forced vital capacity (FVC) <0.70 as measured by spirometry. OSA was defined as symptoms such as excessive daytime sleepiness, recurrent awakenings from sleep, daytime fatigue, and impaired concentration, as well as overnight monitoring demonstrating five or more obstructed breathing events per hour during sleep (apnea/hypopnea index > 5). OS was defined as the simultaneous occurrence of COPD and OSA in the same patient (8, 18, 19). The exclusion criteria were as follows: (1) age > 80 or <18 years old; (2) lack of clinical data; (3) life expectancy less than 1 year; (4) severe COPD patients with noninvasive or invasive respiratory ventilators; (5) severe renal insufficiency (glomerular filtration rate <30 ml/min); (6) previous upper airway surgery for OSA; (7) a history of arrhythmias with a definitive cause (unrelated to the disease under study); and (8) unwillingness to participate in the study. The enrolled cohort was then divided into three groups: (1) OS group, (2) OSA-alone group, and (3) COPD-alone group. The two control groups were pair-matched with the OS group with respect to age and sex at a ratio of 1:3 (Figure 1).

### Data Collection

All data, including medical history, personal history, and laboratory examination, were obtained from the electronic medical record system of the hospital and recorded using a standardized protocol. Pulmonary function tests were performed according to the guidelines of the American Thoracic Society (20), and full-night polysomnography was performed in accordance with those of the American Academy of Sleep Medicine (21). All clinical diagnoses were made by professional staff according to standardized criteria.

### Outcomes Measures

The primary endpoint in this study was to explore the prevalence rates of four specific types of cardiovascular diseases—coronary heart disease (CHD), heart failure, arrhythmia, and pulmonary arterial hypertension (PAH)—and whether there was any difference in prevalence between patients with OS and those with OSA alone or COPD alone. The secondary endpoint was to screen the risk factors for these diseases.

### Statistical Analysis

The Kolmogorov–Smirnov test was used to test the normality of each data distribution. Categorical variables are reported as counts and percentages, whereas continuous variables are reported as the mean (X) and standard deviation. When comparing groups, the Kruskal–Wallis test was used if the data were not normally distributed, and analysis of variance with a *post-hoc* test was used if the data were normally distributed for continuous variables. The chi-squared test was used for categorical variables. When the ratio of variables to missing values was less than 5, the median of quantitative variables or the most common attribute values of qualitative data were used as simple assignments. Univariable logistic regression models were used to characterize OS compared with COPD alone or OSA alone, and multivariable logistic regression was used to explore the risk factors for cardiovascular diseases. Statistical analyses were conducted with SPSS v18.0.0. *P* < 0.05 were considered statistically significant.

## Results

### Characteristics of Study Participants

Of the 6,295 patients initially recruited to the study, 4,412 had COPD as the main diagnosis, and 2047 had OSA. Through a detailed screening, 74 consecutive patients identified to have overlap syndrome were enrolled in the OS group; 222 patients paired-matched for age and sex were enrolled in the OSA-alone group, and the same method was used to form the COPD-alone group (Figure 1).

In this study, the prevalence rate of COPD in OSA patients was 8.01% (164/2047), and the prevalence rate of OSA in COPD patients was 3.72% (164/4412). The clinical characteristics of the study subjects with OS, OSA alone, and COPD alone are described in Table 1. The study cohort was predominantly composed of men (70%), with a mean age of 60.95 ± 9.35 years. The smoking index (*P* < 0.05), body mass index (BMI) (*P* < 0.01), and partial pressure of carbon dioxide (PCO_2_) (*P* < 0.01) were higher, and the partial pressure of oxygen (PO_2_) (*P* < 0.01) and oxygen saturation (SaO_2_) (*P* < 0.01) were lower in subjects with overlap syndrome than in those from the other two groups. The patients with OS had higher serum levels of brain natriuretic peptide (BNP), lactate dehydrogenase (LDH), and neutrophil granulocytes than the patients with OSA alone (all *P* < 0.05). However, the serum troponin levels and the number of patients who smoked or drank alcohol were similar between the three groups (Table 1).

**Table 1:** Characteristics of patients with OS, OSA, and COPD.

	**OS**	**OSA alone**	**COPD alone**	***P*-value**	***P*-value**	***P*-value**
	***n* = 74**	***n* = 222**	***n* = 222**		**OS and OSA**	**OS and COPD**
Age (year)	60.95 ± 9.35	60.72 ± 9.34	60.76 ± 9.30	0.984		
Male, *n* (%)	52 (70)	156 (70)	156 (70)			
BMI (kg/m^2^)	26.89 ± 3.39	23.29 ± 3.45	22.53 ± 3.76	<0.001	<0.001	<0.001
Tobacco use, *n* (%)	17 (22.97)	30 (13.5)	35 (15.77)	0.155		
Smoking index	958.82 ± 128.35	665 ± 444.43	712.57 ± 380.57	0.049	0.03	0.041
Alcohol use, *n* (%)	1 (1.4)	8 (3.6)	2 (9.0)	0.351		
**Laboratory data**						
PO_2_ (mmHg)	59.69 ± 15.06	76.20 ± 17.54	74.66 ± 19.96	<0.001	<0.001	<0.001
PCO_2_ (mmHg)	56.55 ± 14.64	43.94 ± 11.62	47.36 ± 12.65	<0.001	<0.001	0.493
SpO_2_ (%)	86.38 ± 11.74	92.12 ± 9.54	92.87 ± 5.87	<0.001	<0.001	<0.001
D_2_ polymers (mg/L)	1.24 ± 1.50	0.74 ± 1.48	1.50 ± 3.58	<0.001	0.187	0.476
CRP (mg/dl)	15.16 ± 32.79	8.82 ± 37.37	30.79 ± 36.66	<0.001	0.339	0.008
Neutrophil granulocyte (%)	70.40 ± 10.84	63.71 ± 72.14	72.14 ± 12.18	<0.001	<0.001	0.199
TC (mmol/L)	3.94 ± 1.41	3.99 ± 1.14	3.90 ± 0.94	0.65		
TG (mmol/L)	1.55 ± 1.64	1.89 ± 1.33	1.19 ± 0.72	0.006	0.184	0.262
LDL (mmol/L)	2.34 ± 1.07	2.37 ± 0.83	2.04 ± 0.61	0.085		
BNP	1,198.65 ± 2,523.06	434.25 ± 1,316.00	1,147.23 ± 2,930.81	0.017	0.032	0.884
cTnT	0.03 ± 0.03	0.06 ± 0.47	0.10 ± 0.54	0.628		
cTnI	25.43 ± 46.69	11.40 ± 34.51	20.71 ± 38.50	0.298		
CK (U/L)	82.85 ± 108.58	119.53 ± 273.77	86.31 ± 194.15	0.183		
CKMB (U/L)	13.89 ± 5.90	15.41 ± 23.69	14.78 ± 9.70	0.789		
LDH (U/L)	264.19 ± 72.47	213.04 ± 60.11	252.45 ± 111.49	0.000	<0.001	0.329
**Medications**						
ACEI/ARB, *n* (%)	30 (40.5)	106 (47.7)	40 (18.0)	<0.001	0.281	<0.001
β-blockers, *n* (%)	22 (29.7)	95 (42.8)	41 (18.5)	<0.001	0.047	0.04
Nifedipine, *n* (%)	28 (37.8)	52 (23.4)	45 (20.3)	0.009	0.016	0.002
Antisterone, *n* (%)	26 (35.1)	33 (14.9)	36 (16.2)	<0.001	<0.001	0.001
Diuretic, *n* (%)	18 (24.3)	6 (2.7)	45 (20.3)	<0.001	<0.001	0.461
Amiodarone, *n* (%)	1 (1.4)	6 (2.7)	9 (4.1)	0.507		
Warfarin, *n* (%)	3 (4.1)	3 (1.4)	6 (2.7)	0.318		
Rivaroxaban, *n* (%)	5 (6.8)	9 (4.1)	10 (4.5)	0.604		
Aspirin, *n* (%)	29 (38.2)	151 (68.0)	45 (20.3)	<0.001	<0.001	0.001
Clopidogrel, *n* (%)	7 (9.5)	95 (42.8)	28 (12.6)	<0.001	<0.001	0.467
**Comorbidities**
CCI	3.43 ± 1.58	2.85 ± 1.55	3.03 ± 1.31	0.012	0.003	0.043
Hypertension, *n* (%)	45 (60.8)	55 (24.8)	66 (29.7)	<0.001	<0.001	<0.001
SBP (mmHg)	135.18 ± 21.33	138.79 ± 21.52	126.77 ± 19.41	<0.001	0.279	0.016
DBP (mmHg)	82.38 ± 12.80	81.29 ± 13.75	79.93 ± 10.85	0.452		
Diabetes mellitus, *n* (%)	16 (21.6)	67 (30.2)	27 (12.2)	<0.001	0.156	0.051
Fatty liver, *n* (%)	2 (2.7)	24 (10.8)	3 (1.4)	<0.001	0.033	0.602
Stroke, *n* (%)	15 (20.3)	46 (20.7)	23 (10.4)	<0.001	0.93	0.027
PTE, *n* (%)	4 (5.4)	0	1 (0.5)	<0.001	<0.001	0.004
Venous embolism of the extremities, *n* (%)	4 (5.4)	1 (0.5)	7 (3.2)	<0.001	0.015	0.476

*COPD, chronic obstructive pulmonary disease; OSA, obstructive sleep apnea; OS, overlap syndrome; PCO2, partial pressure of carbon dioxide; PO2, oxygen partial pressure; SpO_2_, oxygen saturation; CRP, C-reactive protein; TC, cholesterol; TG, triglyceride; LDL, low-density lipoprotein; cTnI, cardiac troponin I; cTnT, cardiac troponin T; CK, creatine kinase; CKMB, creatine kinase-MB; LDH, lactate dehydrogenase; ACEI, angiotensin-converting enzyme inhibitors; ARB, angiotensin receptor blockers; SBP, systolic blood pressure; DBP, diastolic blood pressure; CCI, Charlson Comorbidity Index; PTE, pulmonary embolism*.

Regarding medications, the number of patients using nifedipine and spironolactone in the OS group was significantly higher than that in the other two groups (*P* < 0.01), and the number of patients using angiotensin-converting enzyme inhibitors/angiotensin receptor blockers and beta-blockers in the OS group was significantly higher than that in the COPD-alone group (*P* < 0.01). The use of medications such as amiodarone, warfarin, or rivaroxaban was not significantly different among the three groups (Table 1).

For comorbidities, the Charlson comorbidity index was significantly higher in the OS group than in the COPD-alone group or OSA-alone group (*P* < 0.05). Six common comorbidities in these cohorts were evaluated: the prevalence of hypertension and pulmonary thromboembolism (PTE) in the OS group was significantly higher than that in the other two groups (*P* < 0.01); the prevalence of stroke in the OS group was higher than that in the COPD-alone group (*P* < 0.05); and the prevalence of venous embolism of the extremities in the OS group was higher than that in the OSA-alone group (*P* < 0.05). However, no significant difference was observed in the prevalence of diabetes among the three groups (Table 2).

**Table 2:** Polysomnographic findings of the study population.

	**OS**	**OSA alone**	***P*-value**
	***n* = 74**	***n* = 222**	
**Severity of OSA**, ***n*** **(%)**			
Mild	29 (39.13)	89 (40.24)	0.891
Moderate	26 (34.78)	38 (17.07)	0.001
Severe	19 (26.08)	95 (42.68)	0.009
AHI	30.67 ± 18.73	23.04 ± 19.51	0.029
meanSpO_2_, %	89.09 ± 4.43	91.61 ± 34.03	0.001
minSpO_2_, %	64.09 ± 20.30	70.66 ± 30.72	0.141
timeSpO_2_ <90%, %	27.92 ± 26.00	25.92 ± 24.76	0.722
**Sleep architecture, %TST**			
Stage 1 sleep	12.98 ± 14.00	11.3 ± 10.02	0.884
Stage 2 sleep	52.23 ± 18.28	58.14 ± 28.72	0.313
Stage 3 sleep	20.22 ± 15.69	19.54 ± 15.78	0.776
Stage REM sleep	9.85 ± 7.63	10.32 ± 7.36	0.761

*OSA, obstructive sleep apnea; OS, overlap syndrome; AHI, apnea–hypopnea index; CT90, percentage of total sleep time with oxygen saturation spent <90%; TST, total sleep time; REM, rapid eye movement*.

### Polysomnography

In the overlap syndrome population, most patients were categorized as having mild/moderate OSA (mild = 39.13%, moderate = 34.78%), whereas, in the OSA-alone group, nearly half of the patients (42.68%) had severe OSA (Table 2). In the sleep testing study, the apnea/hypopnea index was much higher (30.67 ± 18.73 vs. 23.04 ± 19.51, *P* < 0.05), and the mean oxygen saturation (mean SpO_2_; 89.09 ± 4.43 vs. 91.61 ± 34.03, *P* < 0.01) was much lower in the OS group than in the OSA-alone group, whereas the minimum SpO_2_ and sleep architecture were not different between the two groups.

### Pulmonary Function Test

There was no significant difference in the distribution of COPD severity between the OS group and COPD-alone group (*P* > 0.05), and FEV1% predicted was also comparable in both groups (*P* > 0.05). However, the FEV1/FVC ratio was significantly lower in the OS group than in the COPD alone group. Vital capacity and maximum ventilatory volume (MVV), which represent lung volume and pulmonary ventilation function, were lower in patients with overlap syndrome than in those with COPD alone (Table 3).

**Table 3:** Pulmonary function tests and GOLD classification of the study population.

	**OS**	**COPD alone**	***P*-value**
	***n* = 74**	***n* = 222**	
**Severity of COPD**, ***n*** **(%)**			
GOLD 1	8 (10.81)	27 (12.16)	0.475
GOLD 2	24 (32.43)	93 (41.89)	0.994
GOLD 3	22 (29.72)	46 (20.72)	0.488
GOLD 4	20 (27.02)	56 (25.23)	0.287
FEV1/FVC ratio (%)	62.45 ± 25.51	56.14 ± 19.08	0.001
FEV1 (% predicted)	46.29 ± 24.41	48.06 ± 22.70	0.327
VC (%)	2.11 ± 0.70	2.15 ± 0.96	0.012
TV (%)	1.21 ± 0.61	1.29 ± 0.71	0.202
MVV (cmH_2_O/L/S)	38.65 ± 21.88	53.43 ± 37.82	<0.001

*COPD, chronic obstructive pulmonary disease; OS, overlap syndrome; FEV1, forced expiratory volume in 1 s; FVC, forced vital capacity; VC, vital capacity; TV, tidal volume; MVV, maximum ventilatory volume; GOLD, Global Initiative for Chronic Obstructive Lung Disease*.

### Prevalence of Cardiovascular Events

**Figure 2:**
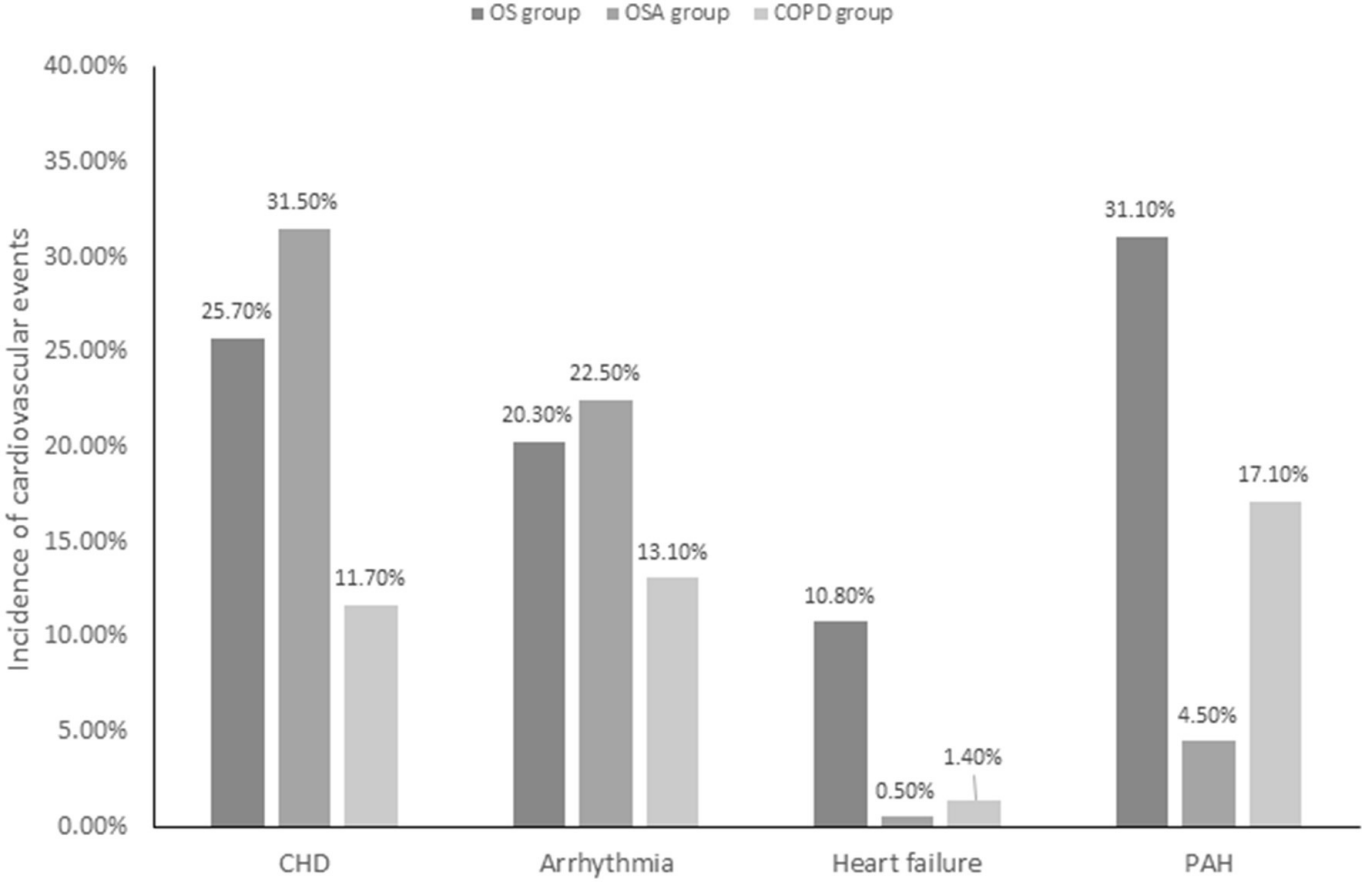
Incidence of cardiovascular events in study patients. OS, overlap syndrome; OSA, obstructive sleep apnea; COPD, chronic obstructive pulmonary disease; CHD, coronary heart disease; PAH, pulmonary arterial hypertension; PTE, pulmonary embolism.

The prevalence rates of four cardiovascular events in the OS, OSA, and COPD groups were 25.7, 31.5, and 11.7% for CHD, 20.3, 22.5, and 13.1% for arrhythmias, 10.8, 0.5, and 1.4% for heart failure, and 31.1, 4.5, and 17.1% for PAH, respectively. The prevalence of heart failure and PAH in subjects with OS was significantly higher than in those with OSA or COPD alone (*P* < 0.01), and the prevalence of CHD in subjects with OS was higher than in those with COPD alone (*P* < 0.01). However, the prevalence of arrhythmias (such as atrial fibrillation, premature atrial contraction, ventricular premature contraction, and atrioventricular or ventricular tachycardia) was not significantly different between the OS group and OSA or COPD groups (Figure 2 and Table 4).

**Table 4:** Incidence of cardiovascular events in study patients.

	**OS**	**OSA alone**	**COPD alone**	***P*-value**	***P*-value**	***P*-value**
	***n* = 74**	***n* = 222**	***n* = 222**		**OS and OSA**	**OS and COPD**
CHD, *n* (%)	19 (25.7)	70 (31.5)	26 (11.7)	<0.001	0.341	0.004
Arrhythmia, *n* (%)	15 (20.3)	50 (22.5)	29 (13.1)	0.011	0.685	0.131
Atrial arrhythmia, *n* (%)	9 (12.2)	23 (10.4)	15 (6.8)	0.253		
AF	8 (10.8)	16 (7.2)	12 (5.4)	0.280		
PAC	1 (1.4)	4 (1.8)	2 (0.9)	0.874		
AT	0	3 (1.4)	1 (0.5)	0.525		
Ventricular arrhythmias, *n* (%)	2 (2.7)	9 (4.1)	4 (1.8)	0.397		
VPB	1 (1.4)	7 (3.2)	2 (0.9)	0.247		
PSVT	1 (1.4)	1 (0.5)	1 (0.5)	0.527		
VT	0	1 (0.5)	1 (0.5)	1		
CRBBB, *n* (%)	1 (1.4)	6 (2.7)	3 (1.4)	0.628		
CLBBB, *n* (%)	0	1 (0.5)	1 (0.5)	1		
SSS, *n* (%)	1 (1.4)	0	1 (0.5)	0.266		
AVB, *n* (%)	0	2 (0.9)	1 (0.5)	1		
Heart failure, *n* (%)	8 (10.8)	1 (0.5)	3 (1.4)	<0.001	<0.001	0.001
PAH, *n* (%)	23 (31.1)	10 (4.5)	38 (17.1)	<0.001	<0.001	0.01
Mild	18 (24.3)	6 (2.7)	15 (6.8)	<0.001	<0.001	<0.001
Moderate	4 (5.4)	3 (1.4)	12 (5.4)	<0.001	0.068	1
Severe	1 (1.4)	1 (0.5)	11 (5.0)	<0.001	0.438	0.306

*CHD, coronary heart disease; AF, atrial fibrillation; PAC, premature atrial contraction; AT, atrial tachycardia; VPB, ventricular premature beat; PSVT, paroxysmal supraventricular tachycardia; VT, ventricular tachycardia; CRBBB, complete right bundle branch block; CLBBB, complete left bundle branch block; SSS, sick sinus syndrome; AVB, atrioventricular block*.

### Risk Factors for Cardiovascular Events

Regarding the risk factors for cardiovascular diseases, multivariate logistic regression showed that hypertension, diabetes, stroke, LDH level, BMI, and tidal volume were positively correlated with CHD prevalence; diabetes, PTE, PCO_2_, PO_2_, MVV, and neutrophil granulocyte percentage were correlated with heart failure prevalence; and PTE, PCO_2_, BNP and LDH levels, and mean SpO_2_ were correlated with PAH prevalence (Table 5).

**Table 5:** Results of logistic regression used to explore risk factors for cardiovascular events.

**Outcomes**	**Variables**	**OR**	**95%CI**	***P*-value**
CHD	Hypertension	3.657	2.262–5.911	0
	Diabetes	1.898	1.151–3.132	0.012
	Stroke	2.459	1.425–4.243	0.001
	LDH	1.004	1.001–1.007	0.003
	BMI	1.963	1.132–3.404	0.016
	TV	0.167	0.084–0.332	0
Heart failure	Diabetes	0.306	0.102–0.916	0.034
	PTE	7.354	1.185–45.635	0.032
	PCO_2_	1.049	1.019–1.080	0.001
	Neutrophil granulocyte	1.053	1.024–1.083	0
	PO_2_	0.975	0.951–0.998	0.036
	MVV	2.341	1.282–4.274	0.006
PAH	PTE	7.191	1.109–46.643	0.011
	PCO_2_	1.046	1.017–1.076	0.002
	BNP	1	1.000–1.000	0.029
	LDH	1.004	1.001–1.007	0.013
	meanSpO_2_	0.063	0.007–0.554	0.013

*CHD, coronary heart disease; PAH, pulmonary arterial hypertension; PTE, pulmonary embolism; LDH, lactate dehydrogenase; BMI, body mass index; TV, tidal volume; MVV, maximum ventilatory volume; CT90, percentage of total sleep time with oxygen saturation spent <90%; PCO_2_, partial pressure of carbon dioxide; PO_2_, oxygen partial pressure; SaO_2_, oxygen saturation*.

## Discussion

In this study, a significant increase in the prevalence of cardiovascular events was observed in the OS population. Patients with OS had a higher prevalence of heart failure and PAH than those with COPD alone or OSA alone (*P* < 0.01), and the prevalence of CHD was also higher in the OS group than in the COPD-alone group (*P* < 0.01). In patients with OS, risk factors for CHD included hypertension, diabetes, stroke, BMI, LDH level, and tidal volume; risk factors for heart failure included diabetes, PTE, PO_2_, PCO_2_, MVV, and neutrophilic granulocyte percentage; and risk factors for PAH included PTE, PCO_2_, minSpO_2_, PCO_2_, and BNP and LDH levels. To the best of our knowledge, this study initially identifies the prevalence and risk factors for cardiovascular events in OS patients in an Asian population compared with both OSA-alone patients and COPD-alone patients.

Although the underlying mechanisms of exactly how OS contributes to cardiovascular events have not been fully elucidated, there are many pathways by which cardiovascular events occur in the presence of OS. The association between the burden of nocturnal hypoxemia, inflammation, and cardiovascular disease in our study is consistent with that found in other clinical investigations in this field (15, 22). In addition, we found that daytime hypoxemia and hypercapnia also contribute to the development of cardiovascular disease.

Hypoxemia is thought to act as a cardiovascular risk factor by increasing cardiac load, inducing oxidative stress by concomitant reactive oxygen species release, impairing vascular endothelial function (23), causing electrophysiological instability in the cardiac conduction system (24), and leading to severe metabolic disorder. Chronic hypoxia and hypercapnia caused by COPD and intermittent nocturnal hypoxia and sleep deprivation caused by OSA can lead to a decrease in the sensitivity of the respiratory center to both hypoxemia and hypercapnia stimulation in patients with OS, leading to further aggravated hypoxemia and hypercapnia. Previous studies confirmed that OS patients have more serious nocturnal hypoxemia, which has been considered a risk factor for cardiovascular diseases (22). However, our study evaluated not only nocturnal hypoxemia but also daytime hypoxemia and hypercapnia, and we found that patients with OS also had higher levels of daytime hypoxemia and hypercapnia than the COPD group or OSA group. Therefore, together with nocturnal hypoxemia, daytime hypoxemia and hypercapnia may have a synergistic effect on promoting cardiovascular diseases. This finding may have clinical implications for developing a better strategy for oxygen therapy in patients with OS.

Hypoxia can upregulate the expression of systemic inflammatory mediators (23, 24) and cause systemic inflammation, which is considered to be another important factor in cardiovascular diseases. There are many common molecular signaling pathways between COPD and OSA, such as C-reactive protein, interleukin 6, and nuclear factor kappa B. Their interaction can cause a systemic inflammatory response and increase body oxidative stress, leading to cardiovascular diseases. Baseline sustained hypoxemia in patients with OS predisposes them to other molecular responses relevant to the mechanisms of cardiovascular disease, especially *via* activation of the transcription factor pathway mediated by hypoxia-inducible factor-1 alpha (25) and related downstream products such as vascular endothelial growth factor. In this study, the level of serum C-reactive protein, a marker of inflammation, was higher in patients with overlap syndrome than in patients with COPD alone, indicating that OSA increased body's inflammatory stress in patients with COPD. There was no significant difference in serum C-reactive protein levels between the OS and OSA-alone groups. One plausible reason is that, in this study, the vast majority of patients in the OSA-alone group were admitted to the hospital for arrhythmias, and these patients already have a higher level of inflammation.

The prevalence of heart failure and PAH in subjects with OS in this study was significantly higher than in those with OSA or COPD alone, and the prevalence of CHD in subjects with OS was higher than in those with COPD alone, which has rarely been reported in real-world studies. No statistically significant difference in the prevalence of arrhythmia between the three groups was found in this study, although there have been studies confirming that patients with OS are at greater risk of AF than those with either COPD or OSA alone (26).

Multivariate logistic regression analysis showed that the prevalence rate of cardiovascular events in this study was correlated with hypertension, diabetes, stroke, PTE, BMI, BNP level, decreased pulmonary function, and nocturnal hypoxemia, which were already believed to be risk factors for cardiovascular diseases (22, 27). In logistic regression analysis, the risk factors for cardiovascular events included daytime hypoxemia and hypercapnia. Therefore, the high incidence of cardiovascular events in patients with OS was speculated to be ultimately caused by hypoxia, hypercapnia, and systemic inflammation. In our study, arterial blood gas analysis was performed in all patients to assess daytime hypoxemia and hypercapnia and is essential in identifying the promoting effect of daytime hypoxemia and hypercapnia on cardiovascular diseases. The benefits of continuous positive airway pressure (CPAP) are now clearly established in patients with OS. Marin and coauthors reported improved long-term survival and a lower rate of hospitalizations in over 200 OS patients treated with CPAP compared with those who were not treated, and the outcomes of those OS patients treated with CPAP were similar to those of patients with COPD alone over a median follow-up period of 9.4 years (28). So, it is reasonable to hypothesize that a more stringent treatment of hypoxia and hypercapnia may be beneficial for patients with overlap syndrome. A prospective, multicenter trial should be carried out to research the effect of improvement of daytime hypoxemia and hypercapnia on cardiovascular outcomes of patients with OS.

We acknowledge certain limitations in this study that should be highlighted when interpreting the findings. First, given the retrospective nature of the study, clinical information on every aspect of cardiovascular events and their risk factors in the cohort was not available; for example, data by Holter, or telemetry monitoring were missing, and therefore, the prevalence of paroxysmal arrhythmias could not be evaluated. Second, this is a single-center retrospective study with a small population. Third, the low incidence of overlap syndrome may lead to an analysis bias, although we enlarged the sample size of the control groups to increase the credibility of the data.

## Conclusion

Patients with OS are at greater risk of cardiovascular events than patients with either COPD or OSA alone, which is related to their impaired pulmonary function, severe hypoxemia, and hypercapnia. It is of great importance for clinical physicians to diagnose and treat patients with OS, promote the management of these patients and improve their quality of life.

## Data Availability Statement

The raw data supporting the conclusions of this article will be made available by the authors, without undue reservation.

## Ethics Statement

The studies involving human participants were reviewed and approved by the Ethics Committee of the First Affiliated Hospital of Xi'an Jiaotong University (No. XJTU1AF2020LSK-187). The patients/participants provided their written informed consent to participate in this study.

## Author Contributions

MT and YL: methodology, writing, and original draft preparation. MT, SL, and XY: data curation and investigation. TS: supervision, writing, reviewing, and editing the manuscript. All authors provided critical review of the manuscript and approved the final draft for publication.

## Conflict of Interest

The authors declare that the research was conducted in the absence of any commercial or financial relationships that could be construed as a potential conflict of interest.

## References

[1] Vogelmeier CF Criner GJ Martinez FJ Anzueto A Barnes PJ Bourbeau J. Global strategy for the diagnosis, management, and prevention of chronic obstructive lung disease 2017 report: GOLD executive summary. Arch Bronconeumol. (2017) 53:128–49. 10.1016/j.arbr.2017.02.001 28274597

[2] Malhotra A Orr JE Owens RL. On the cutting edge of obstructive sleep apnoea: where next? Lancet Respir Med. (2015) 3:397–403. 10.1016/S2213-2600(15)00051-X 25887980 PMC4431916

[3] Lévy P Kohler M McNicholas WT Barbé F McEvoy RDVKS. Obstructive sleep apnoea syndrome. Nat Rev Dis Primers. (2015) 1:15015. 10.1038/nrdp.2015.24 27188535

[4] Jemal A Ward E Hao Y Thun M. Trends in the leading causes of death in the United States, 1970–2002. JAMA. (2005) 294:1255–9. 10.1001/jama.294.10.1255 16160134

[5] Mannino DM Gagnon RC Petty TL Lydick E. Obstructive lung disease and low lung function in adults in the United States: data from the National Health and Nutrition Examination Survey, 1988–1994. Arch Intern Med. (2000) 160:1683–9. 10.1001/archinte.160.11.1683 10847262

[6] Wang C Xu J Yang L Xu Y Zhang X Bai C. Prevalence and risk factors of chronic obstructive pulmonary disease in China. (the China Pulmonary Health [CPH] study): a national cross-sectional study. Lancet. (2018) 391:1706–17. 10.1016/S0140-6736(18)30841-9 29650248

[7] Young T Palta M Dempsey J Skatrud J Webe S Bad S. The occurrence of sleep-disordered breathing among middle-aged adults. N Engl J Med. (1993) 32:1230–5:1230–5. 10.1056/NEJM199304293281704 8464434

[8] McNicholas WT. Chronic obstructive pulmonary disease and obstructive sleep apnea: overlaps in pathophysiology, systemic inflammation, and cardiovascular disease. Am J Respir Crit Care Med. (2009) 180:692–700. 10.1164/rccm.200903-0347PP 19628778

[9] Shawon MS Perret JL Senaratna CV Lodge C Hamilton GS Dharmage SC. Current evidence on prevalence and clinical outcomes of co-morbid obstructive sleep apnea and chronic obstructive pulmonary disease: a systematic review. Sleep Med Rev. (2017) 32:58–68. 10.1016/j.smrv.2016.02.007 28169105

[10] Sin DD Man SF. Chronic obstructive pulmonary disease: a novel risk factor for cardiovascular disease. Can J Physiol Pharmacol. (2005) 83:8–13. 10.1139/y04-116 15759045

[11] Pauwels RA Rabe KF. Burden and clinical features of chronic obstructive pulmonary disease. (COPD). Lancet. (2004) 364:613–20. 10.1016/S0140-6736(04)16855-4 15313363

[12] Park JG Ramar K Olson EJ. Updates on definition, consequences, and management of obstructive sleep apnea. Mayo Clin Proc. (2011) 86:549–54. 10.4065/mcp.2010.0810 21628617 PMC3104914

[13] Epstein LJ Kristo D Strollo PJ Jr Friedman N Malhotra A Patil SP. Clinical guideline for the evaluation, management and long-term care of obstructive sleep apnea in adults. J Clin Sleep Med. (2009) 5:263–76. 10.5664/jcsm.27497 19960649 PMC2699173

[14] Parish JM Somers VK. Obstructive sleep apnea and cardiovascular disease. Mayo Clin Proc. (2004) 79:1036–46. 10.4065/79.8.1036 15301332

[15] Shiina K Tomiyama H Takata Y Yoshida M Kato K Nishihata Y. Overlap syndrome: additive effects of COPD on the cardiovascular damages in patients with OSA. Respir Med. (2012) 106:1335–41. 10.1016/j.rmed.2012.05.006 22705293

[16] McNicholas WT. Diagnosis of obstructive sleep apnea in adults. Proc Am Thorac Soc. (2008) 5:154–60. 10.1513/pats.200708-118MG 18250207

[17] Chaouat A Weitzenblum E Krieger J Ifoundza T Oswald M Kessler R. Association of chronic obstructive pulmonary disease and sleep apnea syndrome. Am J Respir Crit Care Med. (1995) 151:82–6. 10.1164/ajrccm.151.1.7812577 7812577

[18] Culver BH Graham BL Coates AL Wanger J Berry CE Clarke PK. Recommendations for a standardized pulmonary function report. An official american thoracic society technical statement. Am J Respir Crit Care Med. (2017) 196:1463–72. 10.1164/rccm.201710-1981ST 29192835

[19] Berry RB Budhiraja R Gottlieb DJ Gozal D Iber C Kapur VK. Rules for scoring respiratory events in sleep: update of the 2007 AASM Manual for the scoring of sleep and associated events. Deliberations of the sleep apnea definitions task force of the American Academy of sleep medicine. J Clin Sleep Med. (2012) 8:597–619. 10.5664/jcsm.2172 23066376 PMC3459210

[20] MacNee W. Oxidants/antioxidants and COPD. Chest. (2000) 117(5 Suppl 1):303S–17S. 10.1378/chest.117.5_suppl_1.303S-a 10843965

[21] Cortassa S Aon MA Marban E Winslow RL O'Rourke B. An integrated model of cardiac mitochondrial energy metabolism and calcium dynamics. Biophys J. (2003) 84:2734–55. 10.1016/S0006-3495(03)75079-6 12668482 PMC1201507

[22] Kendzerska T Leung RS Aaron SD Ayas N Sandoz JS Gershon AS. Cardiovascular outcomes and all-cause mortality in patients with obstructive sleep apnea and chronic obstructive pulmonary disease (Overlap Syndrome). Annals Am Thoracic Soc. (2019) 16:71–81. 10.1513/AnnalsATS.201802-136OC 30372124

[23] Takabatake N Nakamura H Abe S Inoue S Hino T Saito H. The relationship between chronic hypoxemia and activation of the tumor necrosis factor-alpha system in patients with chronic obstructive pulmonary disease. Am J Resp Crit Care. (2000) 161:1179–84. 10.1164/ajrccm.161.4.9903022 10764309

[24] Zamarron C Garcia Paz V Morete E del Campo Matias F. Association of chronic obstructive pulmonary disease and obstructive sleep apnea consequences. Int J Chron Obstruct Pulmon Dis. (2008) 3:671–82. 10.2147/COPD.S4950 19281082 PMC2650593

[25] Ryan S Taylor CT McNicholas WT. Selective activation of inflammatory pathways by intermittent hypoxia in obstructive sleep apnea syndrome. Circulation. (2005) 112:2660–7. 10.1161/CIRCULATIONAHA.105.556746 16246965

[26] Akinnusi M El-Masri AR Lawson Y El-Solh AA. Association of overlap syndrome with incident atrial fibrillation. Intern Emerg Med. (2021) 16:633–42. 10.1007/s11739-020-02469-y 32803632

[27] Hu W Zhao Z Wu B Shi Z Dong M Xiong M. Obstructive sleep apnea increases the prevalence of hypertension in patients with chronic obstructive disease. COPD. (2020) 17:523–32. 10.1080/15412555.2020.1815688 32901534

[28] Marin JM Soriano JB Carrizo SJ Boldova A Celli BR. Outcomes in patients with chronic obstructive pulmonary disease and obstructive sleep apnea: the overlap syndrome. Am J Respir Crit Care Med. (2010) 182:325–31. 10.1164/rccm.200912-1869OC 20378728

